# Whole body vibration compared to conventional physiotherapy in patients with gonarthrosis: a protocol for a randomized, controlled study

**DOI:** 10.1186/1471-2474-11-128

**Published:** 2010-06-21

**Authors:** Gregor Stein, Peter Knoell, Christoph Faymonville, Thomas Kaulhausen, Jan Siewe, Christina Otto, Peer Eysel, Kourosh Zarghooni

**Affiliations:** 1 Department of Orthopaedic and Trauma Surgery, University of Cologne, Kerpener Strasse 62, 50924 Cologne, Germany; 2 Clinical trials centre, BMBF 01KN0706, University of Cologne, Kerpener Strasse 62, 50924 Cologne, Germany

## Abstract

**Background:**

Osteoarthritis (OA) is the most common degenerative arthropathy. Load-bearing joints such as knee and hip are more often affected than spine or hands. The prevalence of gonarthrosis is generally higher than that of coxarthrosis.

Because no cure for OA exists, the main emphasis of therapy is analgesic treatment through either mobility or medication. Non-pharmacologic treatment is the first step, followed by the addition of analgesic medication, and ultimately by surgery.

The goal of non-pharmacologic and non-invasive therapy is to improve neuromuscular function, which in turn both prevents formation of and delays progression of OA. A modification of conventional physiotherapy, whole body vibration has been successfully employed for several years. Since its introduction, this therapy is in wide use at our facility not only for gonarthrosis, but also coxarthrosis and other diseases leading to muscular imbalance.

**Methods/Design:**

This study is a randomized, therapy-controlled trial in a primary care setting at a university hospital. Patients presenting to our outpatient clinic with initial symptoms of gonarthrosis will be assessed against inclusion and exclusion criteria. After patient consent, 6 weeks of treatment will ensue. During the six weeks of treatment, patients will receive one of two treatments, conventional physiotherapy or whole-body-vibration exercises of one hour three times a week. Follow-up examinations will be performed immediately after treatment and after another 6 and 20 weeks, for a total study duration of 6 months. 20 patients will be included in each therapy group.

Outcome measurements will include objective analysis of motion and ambulation as well as examinations of balance and isokinetic force. The Western Ontario and McMaster Universities Arthritis Index and SF-12 scores, the patients' overall status, and clinical examinations of the affected joint will be carried out.

**Discussion:**

As new physiotherapy techniques develop for the treatment of OA, it is important to investigate the effectiveness of competing strategies. With this study, not only patient-based scores, but also objective assessments will be used to quantify patient-derived benefits of therapy.

**Trial registration:**

Deutsches Register Klinischer Studien (DRKS) DRKS00000415

Clinicaltrials.gov NCT01037972

EudraCT 2009-017617-29

## Background

Osteoarthritis (OA) is the most common degenerative arthropathy. Patients affected by this disease suffer from numerous sequelae, and costs of treatment are high enough to have socio-economic relevance [1-3]. Prevalence of OA rises steadily with advancing age, and women are more typically affected than men [4-6]. Load-bearing joints in particular such as knee and hip are more often affected than spine or hands. Prevalence and incidence of clinical symptoms, and radiologically confirmed gonarthrosis range from 1.6 - 9.4% and 240 out of 100.000 [7,8]. The prevalence of gonarthrosis is usually higher than that of coxarthrosis, particularly in middle-aged patients [9].

Gonarthrosis is commonly defined as a degenerative, non-infectious disease of the knee joint. Morphologically, arthrosis is characterized by progressive loss of cartilage, sclerosis of the subchondral osseous structures, and partial involvement of the synovia [10].

The cardinal symptom of gonarthrosis is pain that is usually activity-related and is intensified by weight-bearing. Initially, the pain has an episodic character and can be relieved by rest, but with disease progression the pain intensifies and becomes more constant.

When patients present with severe pain, motor impairment, or joint effusion, the arthrosis is refered to as activated [9].

Further clinical signs of gonarthrosis are crepitus, palpable osteophytes, coarsening of joint contour, varus or valgus deformity, and joint instability. Because almost 40% of patients with OA are asymptomatic, Kellgren and Lawrence introduced a set of radiologic criteria in 1957. A more specific classification was developed in 1986 by the American Rheumatism Association (ARA), now called the American College of Rheumatology (ACR). These criteria have been validated and include several clinical and radiographic examinations [11]. As long as no cure exists, the main emphasis of OA treatment is analgesia, through either increasing mobility or medication administration. Initially, non-pharmacologic therapies are attempted, followed by the addition of medication, and ultimately surgical intervention [12-14].

For non-pharmacologic and non-invasive therapy, the goal of treatment is to optimize neuromuscular function, which acts both to prevent as well as to delay progression of the disease [15]. Muscle training to correct imbalance, relieve the joints, and invigorate surrounding structures is essential to this therapy. The goals of treatment are twofold--to provide analgesia for patients, but also to delay the need for pharmacotherapy. Because of this, certain enhancements to conventional physiotherapy have been introduced. One such modification is the use of whole body vibration devices, available since 2001. For this, patients stand on a vibrating plate with adjustable amplitude and frequency. The idea is to activate counter-regulatory contractions through the distension of reflexes, which leads to fine-tuning of the neuromuscular spindles. Since its introduction, this therapy has been used widely at our facility not only for gonarthrosis, but also coxarthrosis and other diseases causing muscular imbalance.

Various studies have identified a number of results after the use of whole body vibration. For instance, improvements in muscular strength and coordination as well as an arrest of muscle atrophy have been observed [16,17]. It also improves osseous metabolism [18].

The general assumption is that whole body vibration causes minor stress of the joint affected by OA. Examining women with gonarthrosis, Trans et al. found that whole body vibration led to increased muscle formation and improved proprioception [19]. Male patients have not yet been assessed.

Clearly, an investigation of the use of whole body vibration in men alone would be an interesting clinical study. However, because the success of treatment has so far been measured mostly using questionnaires, we also thought it expedient to introduce validated questionnaires like WOMAC and SF-12, and also assess balance, mobility, and general function of the lower legs. By these means, we should achieve some outlook of this technique from a long-term perspective.

### Objective

The objective of this study is to compare the effects of physiotherapy alone or physiotherapy conducted with a whole body vibration device for the treatment of mild to moderate symptomatic gonarthrosis. The study would be conducted in a primary care setting at a university hospital. Questionnaire assessments relating to clinical outcome as well as objective analysis of ambulation and balance will be performed.

## Methods and Design

The study is designed as a randomized, therapy-controlled trial in a primary care setting at a university hospital. Patients presenting to our outpatient clinic with early-stage gonarthrosis will be assessed against study inclusion and exclusion criteria. After patient informed consent, 6 weeks of therapy will ensue. Follow-up examinations will be performed immediately after treatment and after another 6 and 20 weeks, for a total study duration of 6 months.

Source of funding for the study is the "Deutsche Arthrose Hilfe e.V.", a non-profit patients support group promoting scientific research on osteoarthritis. Funding has been used for the conduction of therapy, sample size calculations, Womac and SF-12 licenses and for equipment acquisition.

Experimental research in this trial will be performed with the approval of the ethics committee of the medical faculty of the University of Cologne under the reference number 10-006. Research carried out in the trial will be in compliance with the Helsinki Declaration.

### Participants and recruitment

Patients aged 30 - 80 years presenting to our outpatient clinic with symptoms of early-stage gonarthrosis are eligible for the trial. Further inclusion criteria are summarized in the appendices.

Patients participating in parallel interventional studies as well as patients suffering from severe gonarthrosis are excluded from this study. The exclusion criteria are summarized in the appendices.

### Interventions

During the six weeks of treatment, patients will receive one of two treatments:

■ Conventional physiotherapy

■ Whole-body-vibration exercises

#### Conventional physiotherapy

Patients in this study group will attend physiotherapeutic exercise sessions of one hour three times a week for six weeks. The sessions consist of aerobic and muscle strengthening as well as coordination exercises. Patients will practice activities of daily living. The goals of these exercises are to improve joint stability, optimize knee and ankle proprioception, and advance neuromuscular innervation of the lower extremity and thereby suppress pathologic motion patterns. This should lead to optimized mobility, increased stability, and thus more endogenous analgesia of the affected joint.

#### Whole body vibration

Patients in this study group will attend whole body vibration exercise sessions of one hour three times a week for six weeks, using the Galileo^® ^Fitness device. Initial training sessions will focus on patient acclimatization, and afterwards improved on muscular capacity and body coordination. During exercise sessions, patients will do 6 training cycles of 3 minutes each. The goals of this treatment are improved proprioception of the ankle and knee joints, as well as optimization of neuronal reactivation of the muscles and thereby improved joint stability. This should also increase endogenous analgesia.

### Outcome measures and assessments

The primary outcome measure is the patients' evaluation of improvement on the visual analogue scale of the WOMAC indices for pain and activities of daily life, comparing baseline and post-treatment. The secondary outcome measures summarized in the appendices will also be assessed at baseline and after 6, 12, and 26 weeks.

The WOMAC index is a validated patient questionnaire used to evaluate coxarthrosis and gonarthrosis by evaluating symptoms and motor impairment in daily life [20,21]. Use of this index in clinical studies is recommended by OARSI and EMEA and has received approval from many studies [20].

Functional motion analysis, the Modified Clinical Test of Sensory Interaction in Balance, the Tandem Walk Test, and the Rhythmic Weight Shift Test will be performed using the Leonardo Mechanography Gangway (Novotec Inc., Pforzheim, Germany) and the Balance Master Analysis System (Neurocom Inc., Clackamas, USA). These will provide reliable data on essential parts of ambulation, e.g. stride length, speed of movement, shifting of balance point, force, power, and workload.

Overall patient status is recommended as a secondary endpoint by EMEA and GREES [11,22]. According to the recommendations of Bellamy et al. [23], study participants will be asked questions regarding symptoms of the treated and other joints as well as subjective health status.

The Osteoarthritis Research Society International (OARSI) and the Outcome Measures in Rheumatology Committee (OMERACT) have developed common criteria to assess patient response to therapy for OA [24,25]. The criteria assess improvements of pain, affected joint function, as well as overall patient status.

The SF-12 score uses subjective patient responses to measure success of therapy and thereby evaluate quality of life. SF-12 is the abbreviated version of the SF-36 Health Survey and contains 12 items representing 8 dimensions of physical and mental fitness. In our study, the validated German translation will be used [26].

The clinical examination of the knee joint will assess intraarticular effusion as well as range of motion.

Finally, the isokinetic force of the knee will be measured using a calibrated "Biodex System 3" (Biodex Medical Systems Inc., Shirley, NY, USA). The maximum isometric force will be measured first by extending and then by flexing the knee.

### Sample size

It is assumed that the standard deviation of the therapeutic effects is σ = 19 mm. Furthermore, no difference between the effects of the different therapy groups is expected. The relevant difference in the scores used for primary outcome is defined as 20 mm. By postulating 80% power using a level of significance of 2.5%, a total number of 31 test patients is needed. When considering a potential drop-out of 4 patients per treatment group, a total of 40 patients will be included into the study.

### Randomization

The randomization of patients into the groups of intervention and control is achieved by using blocks of randomized size. The groups itself are furthermore stratified into gender groups. Technically a locked container with sequentially numbered envelopes is used. The random allocation sequence was generated by the institute of medical statistics, informatics and epidemiology of the university conducting the trial. Enrollment and randomization will be executed by the investigator.

## Discussion

OA remains a major disease in the field of musculoskeletal disorders. Patients presenting with early-stage OA are typically treated with non-curative therapies such as analgesics. Until a cure is found, therapy focused on the roots of symptomatic complaints should be used as well to bridge the gap between disease onset and operative therapy such as partial or total joint replacement. Conventional physiotherapy is well established as one alternative for this bridge. However, new treatment methods like whole body vibration training also seek to improve muscle coordination and thereby optimize affected joint loading. Because both conventional physiotherapy and whole body vibration have co-existed for several years, we can now compare them in an attempt to optimize non-operative treatment for patients with OA.

## Competing interests

The authors declare that they have no conflicting or competing interests in carrying out this study. Deutsche Arthrose-Hilfe e.V., a non-profit patient support organization, will support the study. None of the authors receives or has received any funding from the Deutsche Arthrose-Hilfe e.V. Deutsche Arthrose-Hilfe e.V. has not been involved in the design of the study and will not be involved in analysis of the data and publications.

## Authors' contributions

GS and KZ prepared the study and participated in its design and conduction. GS conceived of the study. All authors revised the manuscript and participated in the conduction of the study. All authors read and approved the final manuscript.

## Appendix

Inclusion criteria:

- Age 30 - 80 years

- Body weight less than or equal to 160 kg

- Body-Mass-Index less than 40 kg/m²

- Outpatient

- Legal competence

- Signed informed consent

- Uni- or bilateral gonarthrosis according to ACR criteria

- WOMAC-pain index (visual analogue scale) of 30-70 mm

- Gonarthrosis stage II-III according to Kellgren and Lawrence

Exlusion criteria:

- Participation in parallel interventional studies

- Bilateral gonarthrosis with WOMAC Pain index ≥ 70 mm

- Dominant femoro-patellar gonarthrosis

- Previous surgery during the past 6 months (exception: meniscus surgery: past 3 months)

- Injury of the study joint during the last 6 months

- Secondary rheumatoid or septic arthrosis or systemic diseases affecting the study joint

- Activated gonarthrosis with intraarticular effusion

- Body weight > 160 kg or body mass index > 40 kg/m²

- Analgesic therapy with steroidal drugs

- Physiotherapy of the lower extremities during the past 6 weeks

- Existing endoprothesis in the lower extremities

Secondary outcome measures:

- Functional motion analysis (Walk Across Test)

- Modified Clinical Test of Sensory Interaction in Balance

- Tandem Walk Test

- Rhythmic Weight Shift Test

- WOMAC^® ^global index

- Overall patient status

- Response using the criteria of OMERACT-OARSI

- Quality of life measured by SF-12^®^

- Clinical examination of the knee joint

- Isokinetic measurement of forces in the knee joint

## Pre-publication history

The pre-publication history for this paper can be accessed here:

http://www.biomedcentral.com/1471-2474/11/128/prepub
